# Efficacy of Eye Movement Desensitization and Reprocessing for Posttraumatic Stress Disorder: A Systematic Review and Meta-Analysis of Randomized Controlled Trials Comparing Passive and Active Control Conditions

**DOI:** 10.7759/cureus.111244

**Published:** 2026-06-21

**Authors:** Ron Gabriel A Peji, Diane D Lipat, Alyssia M De Rojas

**Affiliations:** 1 Center for Psychotraumatology, Livence Mental Health, Silang, PHL; 2 Social Sciences Research, ADP &amp; Co. Research Services and Consultancy, Silang, PHL; 3 Psychology and Human Services, University of Batangas, Batangas City, PHL

**Keywords:** bilateral stimulation, emdr, psychotherapy outcomes, ptsd treatment, randomized trials, symptom reduction, trauma therapy, treatment comparison

## Abstract

Eye movement desensitization and reprocessing (EMDR) is a trauma-focused psychotherapy widely used in the treatment of posttraumatic stress disorder (PTSD), yet its effectiveness across different comparison conditions remains an area of ongoing investigation.

This study systematically reviewed and quantitatively synthesized randomized controlled trials (RCTs) identified through searches of PubMed, Scopus, Web of Science, and PsycINFO for studies published between January 2000 and December 2024, with the final search conducted in August 2025. Study selection was performed through a two-stage screening process by independent reviewers. A total of 11 studies were included in the meta-analysis, which was conducted using a random-effects model. The findings demonstrated that EMDR was associated with a significant reduction in PTSD symptom severity compared with control conditions. Greater effects were observed when compared with passive controls, while effects were smaller and comparable when evaluated against active interventions. Risk of bias was assessed using the Cochrane Risk of Bias (RoB) 2.0 tool, with most studies showing some methodological concerns.

Overall, the evidence supports EMDR as an effective treatment for PTSD and highlights the importance of comparator type in interpreting outcomes.

## Introduction and background

Posttraumatic stress disorder (PTSD) is a pervasive and debilitating mental health condition that poses significant public health challenges worldwide. Characterized by intrusive recollections, hyperarousal, avoidance, and negative alterations in cognition and mood, PTSD is associated with substantial impairment in functioning, reduced quality of life, and increased societal burden [1]. Over the past decades, several trauma-focused psychotherapies have been developed to address these symptoms, among which eye movement desensitization and reprocessing (EMDR) has emerged as a widely endorsed and evidence-based treatment [2]. Nevertheless, despite substantial empirical support and widespread clinical adoption, ongoing debate remains regarding the extent to which EMDR’s therapeutic effects are attributable to bilateral stimulation, trauma-focused exposure processes, or other non-specific psychotherapeutic factors.

A growing body of randomized controlled trials (RCTs) supports the efficacy of EMDR across diverse populations and settings. For instance, EMDR has demonstrated effectiveness not only in adults but also in children and adolescents, highlighting its adaptability across developmental stages [3]. Furthermore, studies have shown its applicability in complex clinical populations, including individuals with comorbid psychiatric conditions [4]. These findings underscore EMDR’s potential as a versatile intervention in both clinical and humanitarian contexts.

Despite its established efficacy, important questions remain regarding the magnitude and consistency of EMDR’s treatment effects, particularly in comparison with other therapeutic approaches. Previous studies have suggested that while EMDR is effective in reducing PTSD symptoms, its relative advantage over other active treatments such as cognitive-behavioral therapy (CBT) or prolonged exposure may be less pronounced [5]. Moreover, variability in study designs, populations, outcome measures, and comparator conditions contributes to heterogeneity in reported findings, complicating efforts to draw definitive conclusions.

In addition, while theoretical models such as the Adaptive Information Processing (AIP) framework have been proposed to explain EMDR’s mechanisms of action [6], alternative perspectives suggest that it effectiveness may also be influenced by non-specific therapeutic factors common to many psychotherapies, including therapeutic alliance, expectancy effects, and structured exposure to traumatic material [7,8]. These considerations highlight the need for a more nuanced evaluation of EMDR’s efficacy that accounts for the type of comparator condition used in clinical trials. Importantly, differences in efficacy observed across comparator types should not be interpreted as direct evidence for any specific mechanism of action, as the present review is designed to evaluate comparative treatment outcomes rather than therapeutic mechanisms.

Although prior systematic reviews and meta-analyses have demonstrated the effectiveness of EMDR for PTSD, there remains a lack of clarity regarding how its efficacy varies when compared with different types of control conditions. In particular, distinguishing between passive controls (e.g., wait-list or no treatment) and active controls (e.g., other evidence-based psychotherapies or pharmacotherapy) is critical for interpreting treatment effects. Passive control conditions generally do not account for non-specific therapeutic influences such as clinician attention, treatment expectancy, and therapeutic alliance, which may inflate observed treatment effects. In contrast, active comparator conditions provide a more rigorous estimate of relative efficacy by controlling for many of these factors. Failure to account for comparator type may lead to overestimation of treatment efficacy and contribute to heterogeneity across studies. Therefore, a focused synthesis of RCTs that explicitly examines these differences is warranted.

This study aimed to evaluate the efficacy of EMDR in reducing PTSD symptom severity through a systematic review and meta-analysis of RCTs. Specifically, the study sought to: (1) estimate the overall effect of EMDR compared with control conditions; (2) examine the magnitude of EMDR’s effect when compared with passive control conditions; and (3) assess its relative efficacy compared with active treatment comparators. By addressing these objectives, this study aims to provide a more precise and clinically meaningful understanding of EMDR’s role in the treatment of PTSD.

## Review

Eligibility criteria

This review included RCTs evaluating the efficacy of EMDR in individuals with PTSD or clinically significant posttraumatic stress symptoms. Eligible studies involved children or adults who met formal PTSD diagnostic criteria based on standardized classification systems (e.g., Diagnostic and Statistical Manual of Mental Disorders-Fourth Edition, Text Revision (DSM-IV-TR) [9], DSM-Fifth Edition (DSM-5) [10], International Classification of Diseases (ICD)-based classifications [11], or who demonstrated clinically significant PTSD symptomatology as defined by validated diagnostic thresholds or subthreshold PTSD classifications used within the original studies. Studies were required to include EMDR as a primary intervention, either as a standalone treatment or as part of a structured protocol, and to report quantitative outcomes related to PTSD symptom severity. Comparators included both passive control conditions (e.g., wait-list, treatment-as-usual, or placebo) and active control conditions (e.g., CBT, prolonged exposure therapy, pharmacotherapy, or other structured psychological interventions). Studies were excluded if they did not include participants with PTSD or clinically significant posttraumatic stress symptoms. For synthesis, studies were grouped based on comparator type into passive control comparisons and active control comparisons to explore potential sources of heterogeneity. This systematic review and meta-analysis was conducted in accordance with the Preferred Reporting Items for Systematic Reviews and Meta-Analyses (PRISMA 2020) guidelines [12]. The review protocol was not prospectively registered. Although all methodological decisions, including eligibility criteria, outcomes of interest, and subgroup analyses, were defined prior to study selection and synthesis, the absence of prospective registration limits independent verification of these decisions and may increase concerns regarding selective reporting or post hoc analytical choices.

Information sources

A comprehensive literature search was conducted across the following electronic databases: PubMed, Scopus, Web of Science, and PsycINFO. The search was limited to studies published between January 2000 and December 2024 to capture contemporary RCTs conducted following the standardization and broader clinical application of EMDR protocols. Although the final search was performed in August 2025, the predefined search period was maintained to ensure consistency in study selection and analysis.

Search strategy

Search strategies were developed using combinations of controlled vocabulary (e.g., MeSH terms) and free- text terms related to EMDR and PTSD. A typical search string used in PubMed was as follows: *(“EMDR” OR “eye movement desensitization and reprocessing”) AND (“posttraumatic stress disorder” OR “PTSD”) AND (“randomized controlled trial” OR “RCT”).*

Filters were applied to include only studies published in English between January 2000 and December 2024. Similar search strategies were adapted for other databases.

Selection process

Study selection was conducted in two stages: (1) title and abstract screening; and (2) full-text review. Two independent reviewers (RGP and ADP) screened all records for eligibility. Full-text articles of potentially relevant studies were then assessed independently by both reviewers against the inclusion and exclusion criteria. Discrepancies were resolved through discussion and consensus. No automation tools were used in the screening process.

Data collection process

Data extraction was performed independently by two reviewers (RGP and ADP) using a standardized data extraction form. Extracted data were cross-checked for accuracy, and discrepancies were resolved through consensus. No attempts were made to contact study authors for missing data. No automation tools were used in the data extraction process.

Data items

The primary outcome was reduction in PTSD symptom severity, as measured by validated instruments such as the Clinician-Administered PTSD Scale (CAPS) [13], PTSD Checklist (PCL-5) [14], Impact of Event Scale-Revised (IES-R) [15], Posttraumatic Stress Symptoms in Children (PTSS-C) [16], and other standardized measures. When multiple outcome measures or time points were reported, post-treatment outcomes were prioritized. If multiple PTSD measures were available, clinician-administered scales were preferred over self-report measures. Additional data extracted included study characteristics (author, year, location, study design), participant characteristics (age, population type, diagnostic criteria), intervention details (number of sessions, duration, protocol), comparator characteristics, and timing of outcome assessments. Where data were incomplete or not directly reported, summary statistics were derived using standard meta-analytic methods. Specifically, when studies reported 95% confidence intervals (CIs) instead of SDs, SDs were calculated using the formula:

$\mathrm{SD} = \frac{\mathrm{Upper\ CI} - \mathrm{Lower\ CI}}{2 \times 1.96} \times \sqrt{n}$

where n represents the sample size of the group.

When multiple outcome measures or time points were available, post-treatment outcomes and clinician-administered measures were prioritized. No additional imputation beyond these standard transformations was performed.

Study risk of bias assessment

Risk of bias was assessed using the Cochrane Risk of Bias 2.0 (RoB 2.0) tool [17] across five domains: (1) bias arising from the randomization process, (2) bias due to deviations from intended interventions, (3) bias due to missing outcome data, (4) bias in measurement of the outcome, and (5) bias in selection of the reported result. Two reviewers (RGP and ADP) independently assessed each included study. Discrepancies were resolved through discussion, and when necessary, consultation with a third reviewer (DDL). Each study was assigned an overall judgment of low risk, some concerns, or high risk of bias. No automation tools were used in the assessment process.

Effect measures

For continuous outcomes, effect sizes were calculated using Hedges’ g, representing standardized mean differences between EMDR and control groups. A negative effect size indicated a reduction in PTSD symptom severity favoring EMDR.

Synthesis methods

Studies were grouped according to comparator type (passive vs active controls) for subgroup analyses. Means, SDs, and sample sizes were extracted for EMDR and comparator groups. When necessary, SDs were derived from reported CIs using established formulas. Effect sizes were calculated as Hedges’ g with corresponding 95% CIs. A random-effects meta-analysis was conducted using the restricted maximum likelihood (REML) estimator to account for between-study variability. Inverse-variance weighting was applied, incorporating both within-study and between-study variance. To improve the accuracy of statistical inference, standard errors and CIs were adjusted using the Knapp-Hartung method. Statistical heterogeneity was assessed using the I^2 ^statistic and τ^2^. For studies with multiple comparator groups, comparator data were combined or appropriately adjusted to avoid double-counting participants where feasible. When studies included both passive and active comparator conditions, a single comparator was selected for the overall analysis to preserve statistical independence and avoid double-counting participants. Passive comparators were preferentially selected because separate subgroup analyses were conducted to evaluate comparator-specific effects. Comparator type was subsequently examined through predefined subgroup analyses comparing passive and active control conditions. Sensitivity analyses were performed by excluding studies with high risk of bias to assess the robustness of the pooled estimates. All analyses were conducted using SPSS (version 31.0.1.0) [18] and verified using standard meta-analytic procedures.

Certainty assessment

The certainty of evidence for the primary outcome (reduction in PTSD symptom severity) was assessed using the Grading of Recommendations, Assessment, Development, and Evaluation (GRADE) approach [19]. This GRADE assessment was conducted following established guidelines for rating certainty of evidence across domains including risk of bias, inconsistency, indirectness, imprecision, and publication bias. Overall, the certainty of evidence was rated as moderate, primarily due to concerns regarding inconsistency and risk of bias. For comparisons with passive control conditions, the certainty of evidence was rated as low to moderate, reflecting substantial heterogeneity and variability across studies. In contrast, evidence comparing EMDR with active control conditions was rated as moderate, supported by consistent findings and low heterogeneity. While EMDR is likely effective in reducing PTSD symptom severity, further high-quality studies may influence the precision and confidence in these estimates.

The total number of participants reported in the summary of findings (Table 1) reflects the inclusion of a single comparator group per study in the overall analysis. For studies with multiple comparator arms, passive control groups were preferentially selected to avoid unit-of-analysis errors and maintain consistency across studies. Subgroup analyses were conducted separately for passive and active comparator conditions, and the corresponding participant counts reflect the distribution of studies within each subgroup. As such, the total sample size is not duplicated across comparisons.

**Table 1:** Summary of findings (GRADE assessment) Certainty of evidence was downgraded based on the following considerations: (1) Risk of bias: Several included studies were rated as having “some concerns,” with a minority classified as high risk; (2) Inconsistency: Substantial heterogeneity was observed in the overall (I² = 75%) and passive control analyses (I² = 78%); (3) Imprecision: CIs were relatively wide in some analyses; (4) Publication bias: Assessment was limited due to the small number of included studies. PTSD: Posttraumatic stress disorder; EMDR: Eye movement desensitization and reprocessing; GRADE: Grading of Recommendations, Assessment, Development, and Evaluation; CI: Confidence interval

Outcome	Comparison	No. of Studies (Participants)	Effect (Hedges’ g, 95% CI)	Certainty of Evidence (GRADE)	Interpretation
PTSD symptom severity	EMDR vs overall control	11 (N = 534)	-0.65 (-1.06 to -0.24)	Moderate	EMDR likely reduces PTSD symptoms, but effect size varies across studies
PTSD symptom severity	EMDR vs passive control	6 (N = 253)	-0.93 (-1.68 to -0.17)	Low-Moderate	Large effect observed, but confidence limited due to high heterogeneity
PTSD symptom severity	EMDR vs active control	5 (N = 281)	-0.26 (-0.51 to 0.00)	Moderate	EMDR shows comparable efficacy to other active treatments

Results

**Figure 1:**
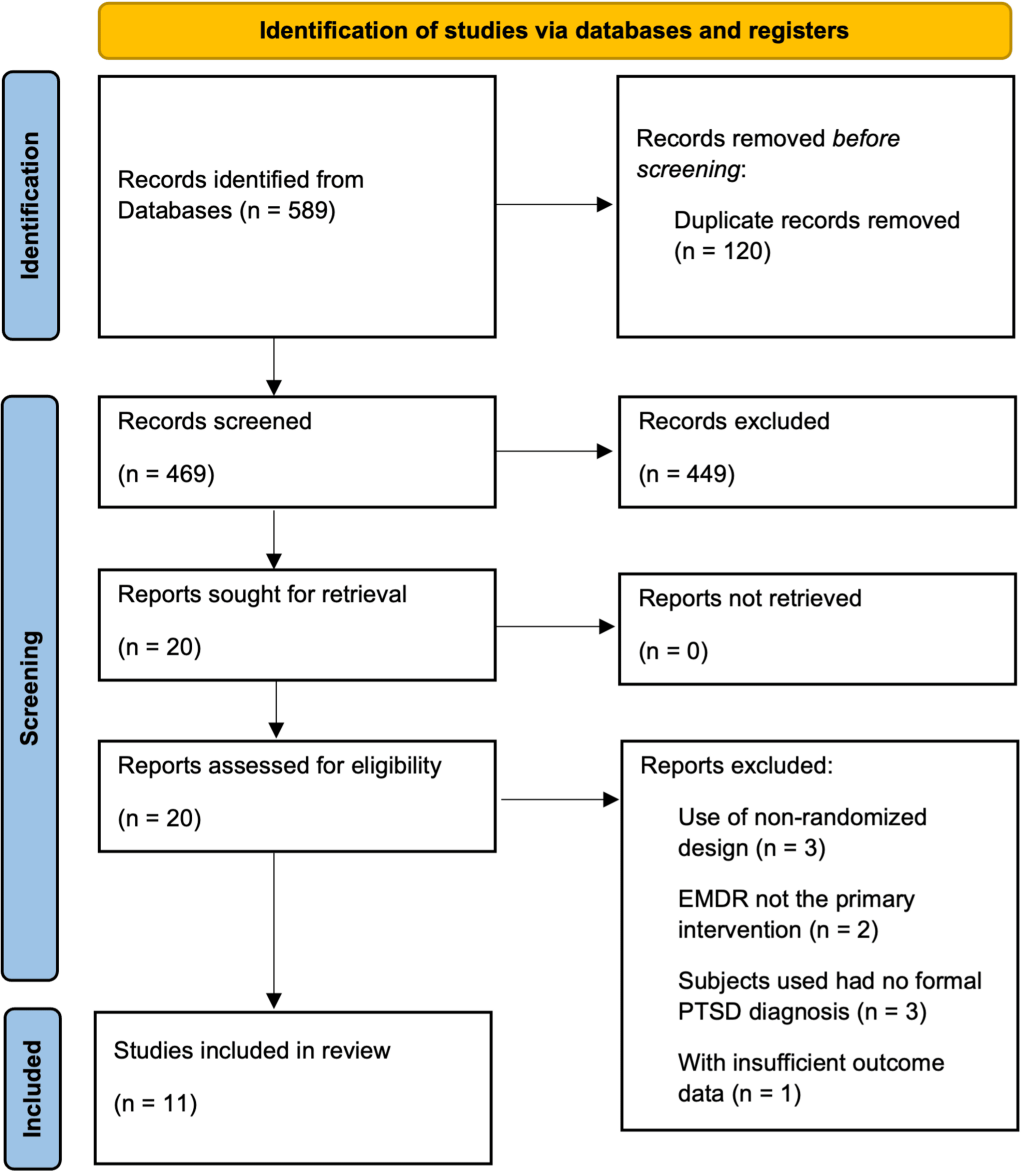
PRISMA flow diagram showing the number of studies from the electronic search up to the final inclusion after thorough screening and multiple evaluations PRISMA: Preferred Reporting Items for Systematic Reviews and Meta-Analyses

A total of 589 records were identified through database searching. After removal of duplicates, 469 unique records remained and were screened based on titles and abstracts. Of these, 449 were excluded for not meeting the inclusion criteria. 20 full-text articles were assessed for eligibility, of which nine were excluded due to non-randomized design (n = 3), absence of EMDR as the primary intervention (n = 2), lack of formal PTSD diagnosis (n = 3), or insufficient outcome data (n = 1). Ultimately, 11 RCTs were included in the qualitative and quantitative synthesis (Figure 1).

Study Characteristics

The characteristics of the included studies are illustrated in Table 2. A total of 11 RCTs, including pilot, feasibility, and multicenter designs, were included in this review, comprising diverse populations across multiple geographic regions, including Turkey, Sweden, the Netherlands, the United Kingdom, and the United States, with one multinational study. Sample sizes ranged from 22 to 155 participants, encompassing both adult and pediatric populations, including refugees, individuals with childhood trauma, community-based trauma survivors, and special populations such as individuals with intellectual disabilities. PTSD diagnosis and inclusion criteria varied across studies, with most employing standardized diagnostic frameworks such as DSM-IV or DSM-5 criteria, while others utilized validated symptom thresholds (e.g., IES-R ≥33 or PCL-5 scores). The EMDR intervention protocols demonstrated variability in intensity and duration, ranging from brief protocols to structured multi-session formats (typically 5 to 12 sessions, lasting 45-90 minutes each), reflecting both standard and adapted implementations of EMDR across different settings. Comparator conditions included both passive controls (e.g., wait-list and placebo) and active interventions (e.g., cognitive behavioral writing therapy (CBWT), prolonged exposure, imagery rescripting, stabilization, standard care, and pharmacotherapy such as fluoxetine), allowing for comprehensive evaluation across treatment contexts. Primary outcome measures predominantly assessed PTSD symptom severity using validated instruments such as the CAPS, IES-R, PTSS-C, and PCL-5, with some studies additionally reporting remission rates or functional outcomes. Follow-up durations varied considerably, ranging from immediate post-treatment assessments to longer-term follow- up periods of up to 15 months, with several studies incorporating intermediate time points (e.g., 3 and 12 months).

**Table 2:** Characteristics of included RCTs evaluating EMDR for PTSD RCT: Randomized controlled trial; EMDR: Eye movement desensitization and reprocessing; PTSD: Posttraumatic stress disorder; IES-R: Impact of Event Scale-Revised; PTSS-C: Posttraumatic Stress Symptoms for Children; CAPS: Clinician-Administered PTSD Scale; CAPS-5: Clinician-Administered PTSD Scale for DSM-5; PCL-5: PTSD Checklist for DSM-5; HTQ: Harvard Trauma Questionnaire; SCID-I: Structured Clinical Interview for DSM-IV Axis I Disorders; PSS-SR, PTSD Symptom Scale - Self-Report; CBWT: Cognitive behavioral writing therapy; VSDT: Visual schema displacement therapy; CR: Cognitive restructuring; DSM-IV: Diagnostic and Statistical Manual of Mental Disorders, Fourth Edition; DSM-5: Diagnostic and Statistical Manual of Mental Disorders, Fifth Edition; DSM-IV-TR: Diagnostic and Statistical Manual of Mental Disorders, Fourth Edition, Text Revision

Study	Country	Design	Sample (N)	Population	PTSD Criteria	Intervention (EMDR)	Comparator	Primary Outcome	Follow-up
Acarturk et al. [20]	Turkey	Pilot RCT	29	Adult refugees	IES-R ≥33 (probable PTSD)	≤7 sessions, 90 min, standard protocol	Wait-list	IES-R	Post, 4 weeks
Ahmad et al. [21]	Sweden	RCT	33	Children (6-16 yrs)	DSM-IV PTSD	8 weekly sessions	Wait-list	PTSS-C	Post
Boterhoven de Haan et al. [22]	Multinational	Multicenter RCT	155	Adults with childhood trauma PTSD	CAPS-5	≤12 sessions, 90 min	Imagery rescripting	CAPS-5	Post, 1 year
de Roos et al. [3]	The Netherlands	Multicenter RCT (3-arm)	103	Youth (8-18 yrs)	DSM-IV PTSD / subthreshold	≤6 sessions, 45 min	CBWT, wait-list	PTSD symptoms	3 & 12 months
Högberg et al. [23]	Sweden	RCT	24	Occupational trauma adults	DSM-IV (SCID-I)	5 sessions, 90 min	Wait-list	PTSD remission	Short-term
Ironson et al. [24]	USA	Pilot RCT	22	Community trauma patients	PSS-SR + clinical eval	Brief EMDR protocol	Prolonged exposure	PTSD symptoms	3 months
Karatzias et al. [25]	UK	Feasibility RCT	29	Adults with intellectual disability	DSM-5 PTSD (PCL-5)	≤8 sessions, ~1 hr	Standard care	PCL-5	3 months
Matthijssen et al. [26]	The Netherlands	RCT (3-arm)	46	Adults with PTSD	DSM-5 (CAPS-5)	6 sessions, 90 min	VSDT, wait-list	CAPS-5	3 months
Power et al. [27]	UK	RCT (3-arm)	105	Adults with PTSD	DSM-IV	≤10 sessions	Exposure + CR, wait-list	CAPS	15 months
ter Heide et al. [28]	The Netherlands	RCT	72	Refugees with PTSD	DSM-IV-TR	9-session structured EMDR	Stabilization	CAPS, HTQ	3 months
van der Kolk et al. [29]	USA	RCT (3-arm)	88	Adults (mixed trauma)	DSM-IV	8-week EMDR	Fluoxetine, placebo	CAPS	6 months

Risk of Bias in Studies

**Figure 2:**
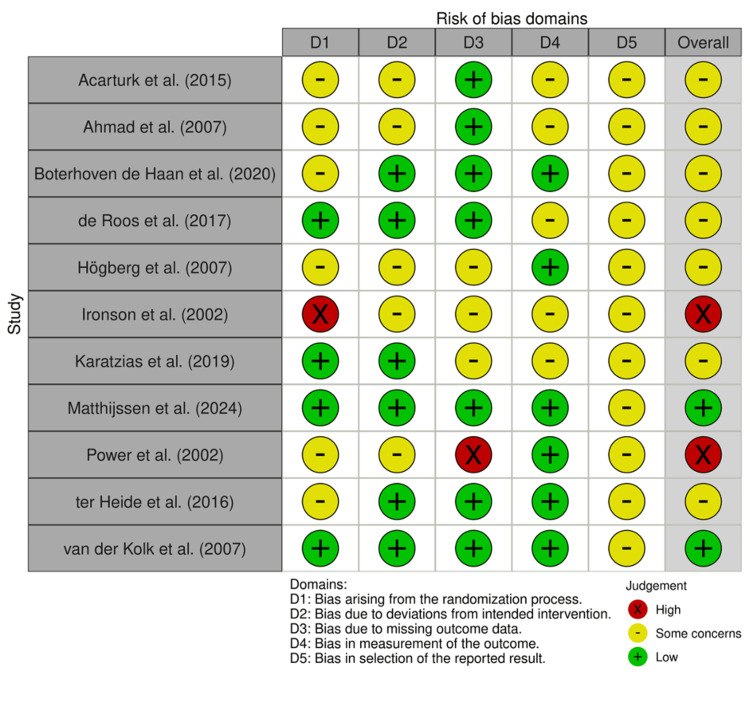
Risk of bias assessment of the included studies

The risk of bias of included studies was assessed using the Cochrane RoB 2.0 tool (Figure 2). Overall, most studies were judged to have “some concerns,” primarily due to the inherent limitations of psychotherapy trials, including lack of participant and therapist blinding and reliance on self-reported outcome measures. Two studies were rated as having a high risk of bias, largely due to issues in the randomization process and handling of missing data. Conversely, two recent trials were judged to have low risk of bias, reflecting improved methodological rigor, including appropriate randomization, blinded outcome assessment, and comprehensive reporting. No studies were excluded based on risk of bias.

Overall Effect of EMDR on Post-treatment PTSD

**Figure 3:**
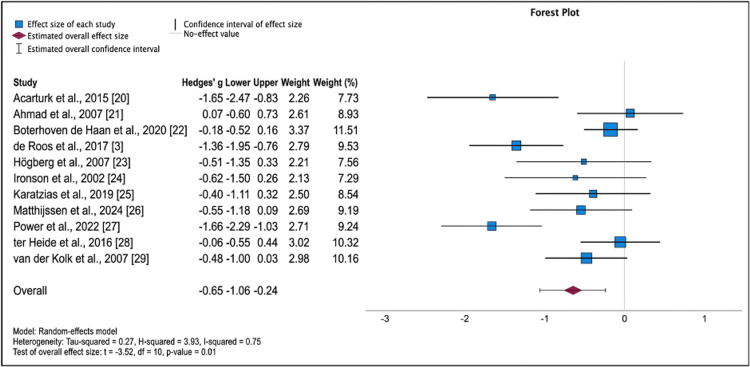
Forest plot of the effect of EMDR on post-treatment PTSD symptom severity compared to control conditions using a random-effects model The numbers in square brackets following the author names correspond to the reference numbers listed in the References section. EMDR: Eye movement desensitization and reprocessing; PTSD: Postttraumatic stress disorder

A random-effects meta-analysis using Hedges’ g was used to determine the overall effect of EMDR on post-treatment PTSD symptoms. For studies with multiple comparator arms, a single comparator group was selected to avoid unit-of-analysis errors. Where applicable, passive control conditions were prioritized for inclusion in the overall analysis to maintain consistency across studies. Findings demonstrated that EMDR was associated with a statistically significant reduction in PTSD symptom severity compared to control conditions (g = -0.65, 95% CI: -1.06 to -0.24, p = 0.01). The magnitude of the pooled effect indicates a moderate-to-large treatment effect favoring EMDR. However, substantial heterogeneity was observed across studies (I^2^ = 75%, τ^2^ = 0.27, p = 0.01), suggesting considerable variability in effect sizes. Visual inspection of the forest plot (Figure 3) revealed that while several studies reported large treatment effects in favor of EMDR, others demonstrated smaller or non-significant differences. This variability may reflect differences in study populations, trauma characteristics, comparator types, and treatment delivery formats.

Despite the overall positive findings, substantial heterogeneity (I^2^ = 75%) was observed, indicating variability in treatment effects across studies. This heterogeneity is likely attributable to methodological and clinical differences, including variations in participant characteristics (e.g., age, trauma type), intervention protocols, comparator conditions (active vs passive), and outcome measures (CAPS, PCL, IES). Importantly, studies employing active comparators (e.g., CBT or pharmacotherapy) tended to demonstrate smaller effect sizes compared to those using passive controls, suggesting that EMDR’s relative efficacy may vary depending on the comparison condition. The findings of this meta-analysis support the use of EMDR as an effective intervention for reducing PTSD symptom severity. The moderate-to-large effect size observed across studies reinforces its role as an effective and evidence-based treatment, particularly in settings where trauma-focused interventions are indicated. However, the variability in outcomes underscores the importance of tailoring treatment approaches based on individual patient characteristics and clinical context.

Sensitivity Analysis

To assess the robustness of these findings, sensitivity analyses were conducted by excluding studies rated as having high risk of bias (i.e., [24,27]). The pooled effect size decreased slightly from Hedges’ g = -0.65 (95% CI: -1.06 to -0.24) to g = -0.53 (95% CI: -0.89 to -0.18), while remaining statistically significant. This finding suggests that although studies with higher risk of bias may have contributed to a modest inflation of the overall effect size, the direction and statistical significance of the treatment effect remained consistent. Heterogeneity was slightly reduced following exclusion (I^2^ = 75% to 70%), indicating that methodological quality may partially account for variability across studies.

EMDR versus Passive Control Conditions on PTSD

**Figure 4:**
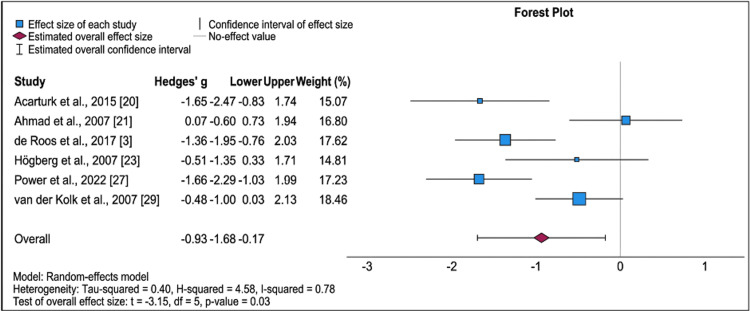
Forest plot of the effect of EMDR versus passive control conditions on PTSD symptom severity The numbers in square brackets following the author names correspond to the reference numbers listed in the References section. EDMR: Eye movement desensitization and reprocessing; PTSD: Posttraumatic stress disorder

A subgroup meta-analysis restricted to studies employing passive control conditions (e.g., wait-list or placebo) demonstrated a statistically significant and large treatment effect favoring EMDR (Figure 4). The pooled effect size was Hedges’ g = -0.93 (95% CI: -1.68 to -0.17, p = 0.03), indicating that participants receiving EMDR experienced substantially greater reductions in PTSD symptom severity compared to those in passive control groups. Despite the strong effect size, considerable heterogeneity was observed across studies (I^2^ = 78%, τ^2^ = 0.40), suggesting variability in treatment effects that may be attributable to differences in study populations, trauma characteristics, and intervention delivery.

The present subgroup analysis indicates that EMDR yields a large and statistically significant reduction in PTSD symptom severity when compared to passive control conditions. This finding is consistent with prior literature suggesting that trauma-focused psychotherapies demonstrate robust effects when contrasted with no-treatment or minimal-intervention conditions.

The larger magnitude of effect observed in this subgroup, relative to the overall pooled estimate, likely reflects the absence of non-specific therapeutic factors-such as clinician attention, expectancy effects, and therapeutic alliance-in passive control conditions. Consequently, the observed effect size may represent a combination of both specific and non-specific treatment effects.

Nevertheless, the presence of substantial heterogeneity indicates that treatment effects are not uniform across studies. Variability in trauma type (e.g., single-incident vs. complex trauma), participant characteristics (e.g., age, clinical severity), and EMDR delivery formats may contribute to this heterogeneity. Future analyses incorporating moderator variables are warranted to further elucidate these differences.

EMDR versus Active Control Conditions on PTSD

**Figure 5:**
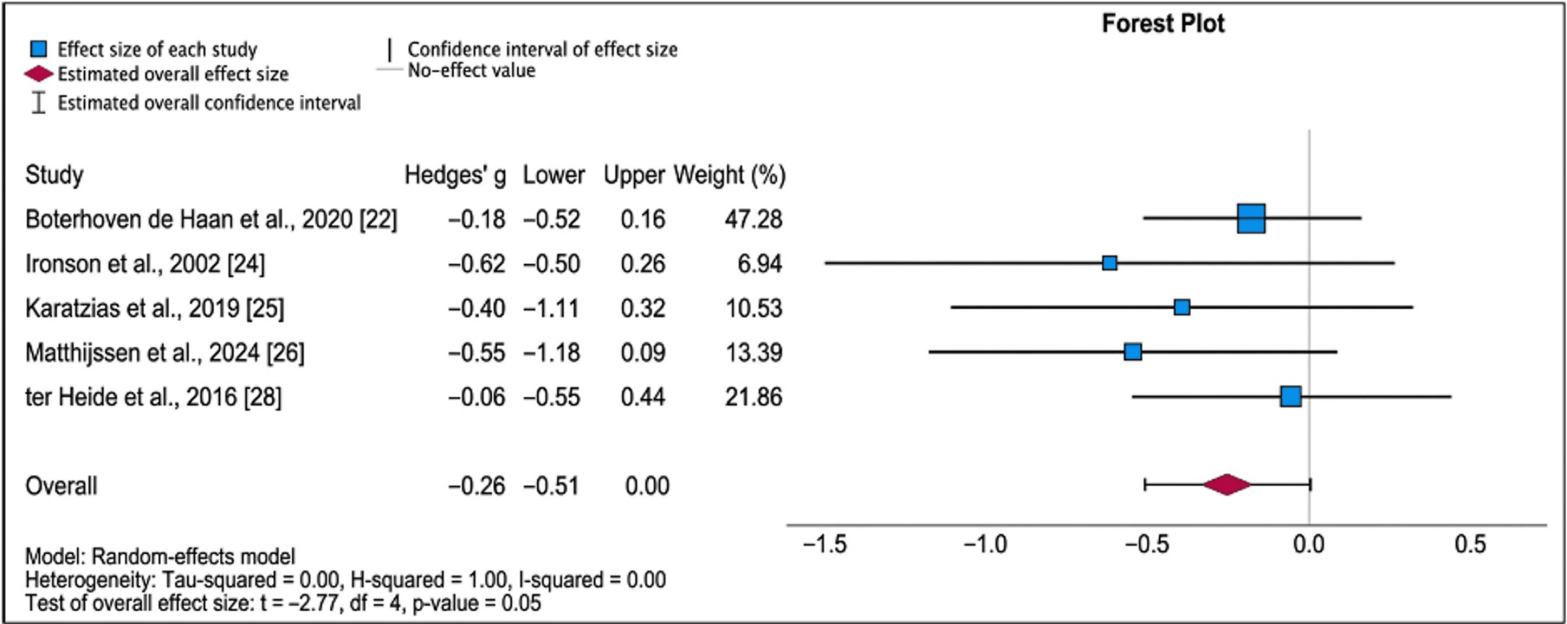
Forest plot of the effect of EMDR versus active control conditions on PTSD symptom severity The numbers in square brackets following the author names correspond to the reference numbers listed in the References section. EDMR: Eye movement desensitization and reprocessing; PTSD: Posttraumatic stress disorder

Furthermore, a subgroup meta-analysis restricted to studies employing active control conditions (e.g., CBT, prolonged exposure, stabilization, or standard care) demonstrated a small-to-moderate treatment effect favoring EMDR (Figure 5). The pooled effect size was Hedges’ g = -0.26 (95% CI: -0.51 to 0.00, p = 0.05), indicating a borderline statistically significant advantage of EMDR over active comparators. Notably, heterogeneity across studies was negligible (I^2^ = 0%, τ^2^ = 0.00), suggesting consistent findings across trials when EMDR is compared with other active interventions.

In contrast to the findings observed in the passive control subgroup, the effect size of EMDR was substantially attenuated when compared with active treatment conditions. This suggests that while EMDR is effective in reducing PTSD symptom severity, its relative advantage over other evidence-based or structured interventions is modest.

The near-zero heterogeneity observed in this subgroup indicates a high degree of consistency across studies, reinforcing the robustness of this finding. The reduced effect size is likely attributable to the presence of non-specific therapeutic factors in active comparators, such as structured intervention, therapist engagement, and expectancy effects, which are absent in passive control conditions.

These findings suggest that EMDR performs comparably to other established treatments for PTSD rather than demonstrating clear superiority. This aligns with current clinical guidelines that position EMDR alongside other trauma-focused psychotherapies as a first-line treatment option.

The marked difference in effect sizes between passive and active comparator subgroups suggests that the magnitude of EMDR’s efficacy is strongly influenced by the nature of the comparison condition. Larger effects observed against passive controls likely reflect both specific treatment effects and non-specific factors, whereas comparisons with active treatments provide a more conservative estimate of EMDR’s relative efficacy.

Discussion

The present meta-analysis provides updated evidence that is broadly consistent with the efficacy of EMDR in reducing PTSD symptom severity across RCTs. The overall pooled effect size indicated a moderate-to-large reduction in PTSD symptoms, with the largest effects observed when EMDR was compared to passive control conditions. These findings are consistent with prior meta-analytic evidence demonstrating that EMDR is a robust and effective trauma-focused intervention [30,31].

Notably, subgroup analyses revealed a differential pattern of effectiveness depending on comparator type. EMDR demonstrated a stronger and statistically significant effect when compared to passive controls (e.g., waitlist or placebo), whereas its effect relative to active comparators (e.g., CBT, exposure-based approaches, or stabilization interventions) was smaller and approached the threshold of statistical significance. This pattern aligns with existing literature suggesting that while EMDR is highly effective, its outcomes appear broadly comparable to those observed with other evidence-based trauma-focused therapies [32,33]. The current results extend this evidence by quantitatively demonstrating that the magnitude of EMDR’s effect is partially contingent on the nature of the control condition. Larger effects observed relative to passive control conditions may reflect not only the specific therapeutic mechanisms of EMDR but also nonspecific factors common to psychotherapy trials, including therapist attention, treatment expectancy, and structured therapeutic engagement.

From a theoretical standpoint, the observed effectiveness of EMDR across studies is consistent with the AIP model, which posits that trauma-related symptoms arise from maladaptively stored memories that can be reprocessed through bilateral stimulation [34,35]. The consistent symptom reduction observed in this meta-analysis is consistent with hypotheses regarding the potential contribution of mechanisms such as working memory taxation, memory reconsolidation, and enhanced emotional regulation, all of which have been discussed in the broader EMDR literature [36,37].

However, the presence of moderate to substantial heterogeneity, particularly in the overall and passive-control analyses, suggests that treatment effects vary across studies. This variability is likely attributable to differences in participant characteristics (e.g., age, trauma type), intervention protocols (e.g., number of sessions, session duration), and study designs, as previously identified in the literature [38,39]. The subgroup analysis based on comparator type partially accounted for this heterogeneity, highlighting the importance of methodological stratification in meta-analytic studies of psychotherapy.

Clinically, the findings of this study underscore EMDR’s value as an effective and versatile intervention for PTSD across diverse populations, including children, refugees, and individuals with complex trauma histories. This is consistent with prior research demonstrating EMDR’s adaptability and applicability across different clinical contexts [3,40]. Furthermore, EMDR’s relatively shorter treatment duration and reduced reliance on verbal processing may enhance its acceptability and feasibility in both high-resource and low-resource settings, alongside other evidence-based trauma-focused interventions [41].

Nevertheless, the results should be interpreted in light of several limitations. Variability in study quality, as reflected in the risk of bias assessment, and inconsistencies in outcome measures and follow-up periods may affect the precision and generalizability of the pooled estimates. Furthermore, the review focused primarily on post-treatment PTSD symptom severity and did not evaluate remission rates, functional outcomes, or minimal clinically important differences, as these outcomes were not consistently reported across the included studies. Some included studies enrolled participants with probable or subthreshold PTSD rather than formal diagnostic PTSD, which may have contributed to clinical heterogeneity and should be considered when interpreting pooled estimates. The review protocol was not prospectively registered, which limits independent verification that eligibility criteria, outcomes, and analytical decisions were fully specified prior to study selection and analysis. In addition, the relatively small number of included studies limited the ability to meaningfully assess publication bias, and the search strategy was restricted to published studies indexed within the selected databases without inclusion of grey literature sources. Sensitivity analyses further demonstrated that exclusion of studies with high risk of bias resulted in a slight attenuation of the pooled effect size, although statistical significance was maintained. This suggests that while methodological quality may have modestly influenced effect estimates, the overall findings remain robust. Additionally, the absence of extensive moderator analyses limits the ability to identify specific conditions under which EMDR is most effective, a gap that has been repeatedly highlighted in the literature [42,43].

## Conclusions

EMDR was associated with significant reductions in PTSD symptom severity, particularly when compared with passive control conditions such as wait-list or placebo, while demonstrating broadly comparable effectiveness to other active trauma-focused interventions. These findings support the use of EMDR as an effective and evidence-based treatment option for PTSD and highlight the importance of comparator type when interpreting treatment effects. However, the variability in effect sizes across studies underscores the influence of methodological and clinical factors, including differences in comparator type, intervention protocols, and participant characteristics. The presence of heterogeneity and varying levels of risk of bias further warrants cautious interpretation of pooled estimates. Future research should focus on conducting high-quality, adequately powered randomized trials with standardized outcome measures and longer follow-up periods to better elucidate the long-term effectiveness of EMDR and its comparative effectiveness relative to other evidence-based interventions.
